# Stress Estimation through Deep Rock Core Diametrical Deformation and Joint Roughness Assessment Using X-ray CT Imaging

**DOI:** 10.3390/s20236802

**Published:** 2020-11-28

**Authors:** Hanna Kim, Melvin B. Diaz, Joo Yeon Kim, Yong-Bok Jung, Kwang Yeom Kim

**Affiliations:** 1 Korea Institute of Geoscience and Mineral Resources, 124, Gwahak-ro Yuseong-gu, Daejeon 34132, Korea; hnkim@kigam.re.kr (H.K.); ybjung@kigam.re.kr (Y.-B.J.); 2 Department of Energy & Resources Engineering, Korea Maritime and Ocean University, 727, Taejongro, Yeongdo-gu, Busan 49112, Korea; jykim@g.kmou.ac.kr

**Keywords:** stress estimation, diametrical core deformation analysis (DCDA), joint roughness, X-ray

## Abstract

In-situ stress estimation plays an important role on the success of an underground project. However, no method is error-free, and therefore a combination of methods is desirable. In this study, the in-situ stresses for a geothermal project have been assessed through the analysis of a deep rock core taken at 4.2 km, using the diametrical core deformation analysis (DCDA) method that relates the diametrical core expansion after stress relief with the stresses assuming elastic deformation. The extracted granodiorite core sample of 100 mm of diameter was intersected with a closed joint at a dip angle of 80.8° with respect to the vertical coring direction. The core sample was scanned using an industrial X-ray computed tomography (CT), and the diametrical deformation measurements were computed with CT slices. Results from using the DCDA method indicated an average horizontal stress difference of 13.3 MPa, similar to that reported for a nearby exploration well. Furthermore, the stress orientations were compared with the orientation of maximum roughness values. The results indicated a correlation between the orientation of the maximum horizontal stress and the orientation of the minimum joint roughness coefficient, implying a possible tracking of stress orientation using joint roughness anisotropy.

## 1. Introduction

Several studies have proposed different methods to assess in-situ stresses. Hydraulic fracturing, flat jack, and the over coring have been typically used to estimate stress magnitudes and orientations. When it comes to measure stresses at a drilling site, hydraulic fracturing becomes the preferable method. However, employing this method often imposes economic constraints. Then, an alternative is to estimate the stresses from recovered rock cores.

Typical methods for stress estimation using rock cores include acoustic emission (AE), anelastic strain recovery (ASR), different strain curve analysis (DSCA), and development rate analysis (DRA). The AE method was proposed in 1950, and it makes use of the so-called Kaiser effect, which characterizes the AE increase under increasing stress. These methods require that the applied stress exceeds that normally experienced by rock cores, and that the drilling of smaller cores occurs in multiple directions. The maximum stress from the selected rock sample can be determined at the point where a significant increase in acoustic emission occurs [1,2,3].

The ASR method is based on the fact that when a core is taken from the rock mass, it tends to expand due to the removal of stresses, a process that is followed by the opening and propagation of microcracks. This method measures the strain recovery of a rock core through a special instrument, and then it determines the orientation of the principal stresses [3]. However, a different study showed that the ASR method often fails to report reliable results, as multiple factors can induce errors. For example, distortion due to temperature changes, dehydration of core samples, diffusion of pore fluid pressure, non-uniformity of recovery displacement, anisotropy of rock, interaction between drilling water and rock, residual deformation, core recovery time, and accuracy of core orientation, can induce errors [4].

In the DSCA method, the strain behavior of rocks upon re-loading serves as a basis to infer the past stress history. After the drilling core is extracted to the surface, fine cracks develop and align with the original stress direction. The strain due to the closure of these microcracks is induced after subjecting the core to hydrostatic pressure, and it is measured through strain gauges [3]. Strickland and Ren [5] confirmed the reliability of in-situ stress estimation using the DSCA method based on calculations of three-dimensional stress conditions. They developed mathematical solutions to obtain stress orientations and their ratios. However, this method is not valid when rocks have been subjected to very high stresses with a high occurrence of microcracks, and also when rocks have a high cohesion that reduces its relaxation and therefore interferes with the stress estimation [6].

Finally, the DRA method estimates the initial stress based on the gradient change for a strain difference function, which is defined as a function of the axial stress, and is obtained by subtracting the axial strain from the first loading from three successive loadings [7]. Another study modeled the AE behavior of a synthetic cylindrical rock model subjected to uniaxial compression, using a discrete element method [8]. They reproduced the Kaiser effect during loading and made a relation between the AE and the DRA methods.

Therefore, although these methods of in-situ stress measurement using rock cores are simple to use and easy to perform in the laboratory regardless of the depth of the cores, there is a disadvantage with relatively low reliability, since the stresses are assessed based on changes in the micro-scale. In this context, an alternative core method could overcome some of these shortcomings, as it proposes to estimate the stresses based on the diametrical core deformation analysis (DCDA) [9]. In short, when a rock core is retrieved in situ, the stresses acting perpendicular to the coring direction induce diametrical deformation that is proportional to their magnitudes due to stress relief. Then, knowing the original and final diameters, as well as the Young’s modulus and Poisson’s ratio, and assuming elastic deformation, the stress difference between horizontal stresses can be computed.

In this study, the in-situ stresses for a geothermal site were assessed using a rock core retrieved at 4.2 km depth. The stresses were estimated using the DCDA method and the diametrical core deformations were calculated through X-ray CT imaging. Also, the orientation of stresses obtained from the core sample and the orientation of roughness anisotropy were compared and discussed. Finally, these results are to be considered as preliminary one, as more tests with multiple samples are desired.

## 2. Background, Materials, and Methods

### 2.1. Description of EGS Site and Retrieved Rock Cores at Great Depth

**Figure 1:**
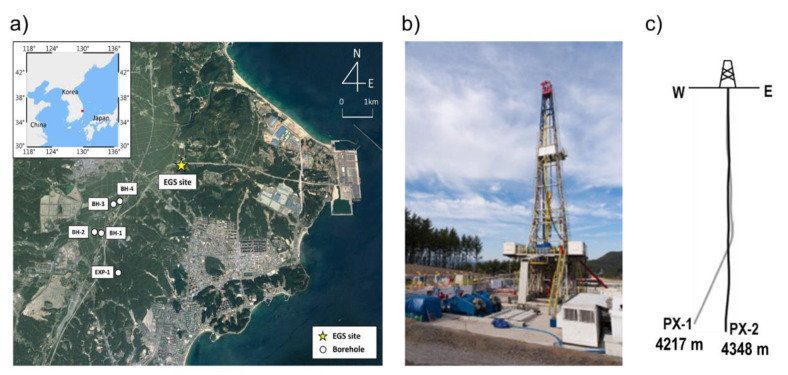
(**a**) Pohang Enhanced Geothermal System (EGS) project location. (**b**) Photo of a well drilling rig, and (**c**) schematic of wells PX-1 and PX-2 depths.

Despite not being a country with high enthalpy for geothermal resources, South Korea started geothermal investigations in 2003 [10], and later in 2010 launched its first Enhanced Geothermal System (EGS) pilot project at the coastal city of Pohang (Figure 1a) [11]. Multiple studies were carried out at that site, starting with geological models [10], stress estimation [12], well drilling related assessments [13,14,15,16], characterization of target rock properties [17,18], and hydraulic stimulations [19,20,21]. Two wells were drilled and named PX-1 and PX-2, with depths of 4127 m and 4348 m, respectively (Figure 1c). However, PX-1 was later side-tracked at an angle of 22° and reached a true vertical depth of 4215 m [16]. The main stratigraphic sections found during PX-1 drilling were composed of mudstone (up to 216 m), combinations of sandstone, mudstone and tuff (up to 2356 m), and granodiorite (up to the target depth 4127).

**Figure 2:**
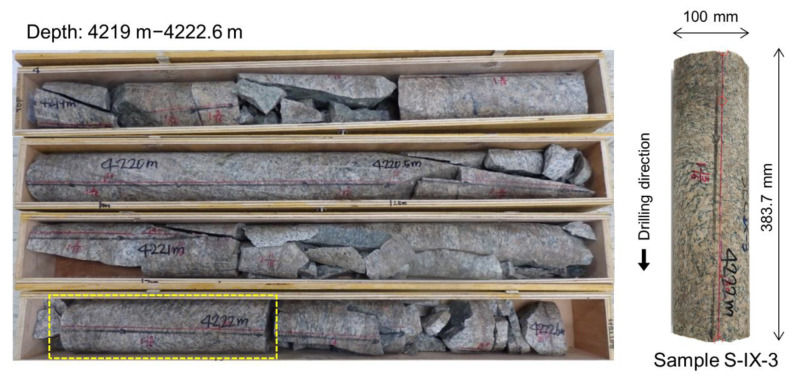
Rock samples extracted from a PX-2 well. The dashed rectangle indicates the studied core sample (S-IX-3) for stress estimation and roughness assessment of a closed joint.

Rock core samples were retrieved from well PX-2 at Pohang EGS pilot site at a depth of 4219 m, with a diameter of 100 mm and covering a depth section of 3.6 m (Figure 2). The cores were categorized into 11 sections based on their conditions (S-I to S-XI). The granodiorite cores served to determine multiple mechanical and thermal properties [17]. X-ray diffractometer and polarization microscope techniques were used to determine the mineral composition and distribution. They revealed a composition of 43.1% albite, 28.6% quartz, 13.7% microcline, and 10.1% muscovite. The elastic modulus, Poisson’s ratio, uniaxial compressive strength, and tensile strength were reported to be 33.5 GPa, 0.21, 106.7 MPa, and 9.2 MPa, respectively [17]. Although granitic rocks commonly present physical anisotropic properties, this feature was not studied from the rock cores extracted at Pohang. Other tests performed with these rock cores included Schmidt hammer test, direct shear test, normal fracture stiffness, shear fracture stiffness, dilation angle, specific heat, thermal conductivity, and thermal expansion. In this study, the core sample at section S-IX was used to estimate in situ stresses through the estimation of diametrical core deformation (Figure 2), and joint roughness anisotropy. Table 1 summarizes the steps followed from site recovery to stress and roughness measurement and interpretation.

### 2.2. Stress models for Pohang Site

Stress models of Pohang EGS site have also been presented. An integrated in-situ stress model was suggested using hydraulic fracturing, borehole observations, and numerical modeling at the exploration well EXP-1 that is 4 km south-west of the EGS site, which reported an in-situ stress through stress ratio. A stress ratio is an alternative way to report stresses taking the vertical stress as a reference and providing the values for maximum and minimum horizontal stresses as the ratio between the measured stress and the vertical stress. The stress ratios for Pohang were reported as 1.3/1.0/0.8 (*S_Hmax_/S_v_/S_hmin_*), with the *S_Hmax_* orientation falling into the range of N130°E–N136°E [12].

A later stress model reported by the Korean Government Commission on Relations between the 2017 Pohang Earthquake and EGS project included a reverse stress state evaluated at 4.2 km depth, with *S_Hmax_*, *S_hmin_*, and *S_v_* being 243 MPa, 120 MPa, and 106 MPa, respectively [22]. They also reported an alternative model based on regional focal mechanisms, and estimated a strike-slip regime with *S_Hmax_*, *S_hmin_*, and *S_v_* being 203 MPa, 93 MPa, and 106 MPa, respectively. The *S_Hmax_* orientation was estimated to be at an azimuth of 77° and 74° for the first and second (regional focal mechanisms analysis) stress models.

### 2.3. Stress Estimation from Diametrical Rock Core Deformation

**Figure 3:**
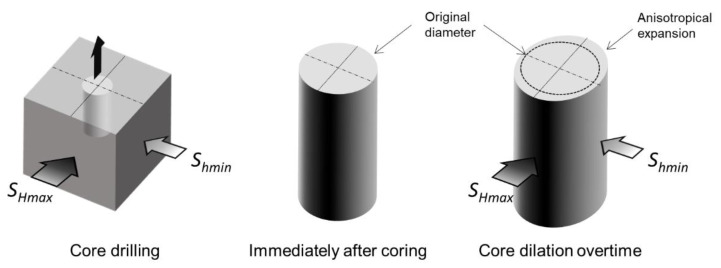
Schematic of the diametrical core deformation analysis (DCDA) method for which a core obtained from a rock block subjected to differential stresses will present diametrical deformation (expansion) which is proportional to the magnitude and orientation of the stresses.

Funato and Ito [9] proposed a method to estimate in-situ stresses based on the diametrical deformation of rock cores following stress relief, and called it the diametrical core deformation analysis (DCDA) method. They stated that after coring, even for homogenous and isotropic rocks should experience diametrical anisotropic expansion in line with the horizontal in-situ stresses (Figure 3).

The rock should expand proportionally to the magnitude of the stress. In other words, with a coring direction perpendicular to the in-situ horizontal stresses, the core sample expands the most and the least in the directions of maximum and minimum horizontal stresses, respectively. They estimated the difference between maximum (*S_max_*) and minimum (*S_min_*) stress, based on the circumferential variations or maximum (*d_max_*) and minimum (*d_min_*) diameters after expansion, while assuming that elastic deformation occurs. In addition, the diametrical core measurements were carried out under no-load conditions using a specifically developed laser scanner. Finally, they summarized this concept in Equation (1), where *d_max_* and *d_min_* are combined with Poisson’s ratio and Young’s modulus to estimate the stress difference:

(1) $$S_{max}-S_{min}=\;\frac{E}{1+v}\frac{d_{max}-d_{max}}{d_{0}}\approx \;\frac{E}{1+v}\frac{d_{max}-d_{max}}{d_{min}}$$

where *E* is Young’s modulus, *v* is Poisson’s ratio, and *d*_0_ is replaced by *d_min_* assuming a small deformation resulting from the stress relief.

Furthermore, Funato and Ito tested this concept with granite core samples extracted at 445–659 m, and with mortal samples at the laboratory. For those core samples, the stress difference estimations were complemented with hydraulic fracturing results to obtain the maximum and minimum stresses. Also, the laboratory results for cubic mortar samples showed a good agreement between the measured and estimated values, thereby indicating the validity of the method.

Motivated by Funato and Ito’s results, in this study, we obtained the diametrical core deformation for the sample S-IX-3 using X-ray CT images, and present the results in the next section. The process of diametrical deformation measurement was straightforward. During scanning, the sample was aligned vertically to make sure that each cross-section or CT slice was perpendicular to the coring direction. The estimation of diametrical deformation was carried out using ImageJ software, an open-source image processing program developed by the National Institutes of Health and the laboratory for optical and computational instruments of the University of Wisconsin [23,24]. For a given CT slice, the first step consisted of binarizing the image. Second, the “wand” tool allowed selecting the contour of the rock core only. Finally, the “measure” command was used to calculate the best-fitted ellipse, and to return the values for major and minor axis, as well as the orientation of the major axis.

### 2.4. Roughness Anisotropy of Rock Joints

A joint roughness assessment on deep cores from Pohang EGS site has also been carried out [18]. As explained in the previous section, granodiorite cores were extracted from well PX-2. These cores are crossed by multiple joints, but can be grouped in two main sets of open and closed joints. However, only the set ranging from 10° to 30° from the coring direction was used to measure joint roughness. The core samples were scanned at the industrial X-ray CT facility of the Korea Institute of Civil Engineering and Building Technology [25]. The scanning conditions were selected according to the sample geometry. For example, the source object distance was different for every case, ranging from 100 to 245.6 mm, as well as the pixel pitch that ranged from 0.06 to 0.17 mm. The typical voltage during scanning was 240 kVp, the current was 500 mA, the exposure time was 1 s, and the number of projections was 1800. After CT scanning, 3D surface models were constructed to later provide point cloud data (x, y, z triads) of the core surface. Moreover, this process allowed for selecting any region of interest such as open or closed joint surfaces.

Diaz et al. [18]’s joint roughness analysis reported two important findings: the roughness anisotropy of each joint and the similar orientation of maximum joint roughness coefficient (*JRC*). This was possible because they measured *JRC* through multiple cross sections (spaced every 2.5 mm) and for multiple orientations at every 10°. Then, to obtain the maximum and minimum *JRC* values, they fitted an ellipse to the *JRC* values per the orientation. What was interesting is that these maximum *JRC* values reported similar orientations ranging from 152.9° to 166.8° measured counterclockwise from the horizontal axis on each joint surface, for both open and closed joints. On the other hand, the average *JRC* values for all joints ranged from 8.7 to 17.

In this job, the roughness of the closed joint CJ-2 was re-assessed, which is present in sample S-IX-3. The orientations of *JRC_max_* and *JRC_min_* are reported in the next section.

## 3. Results and Discussion

### 3.1. Diametrical Core Deformation and Stress Estimation

**Figure 4:**
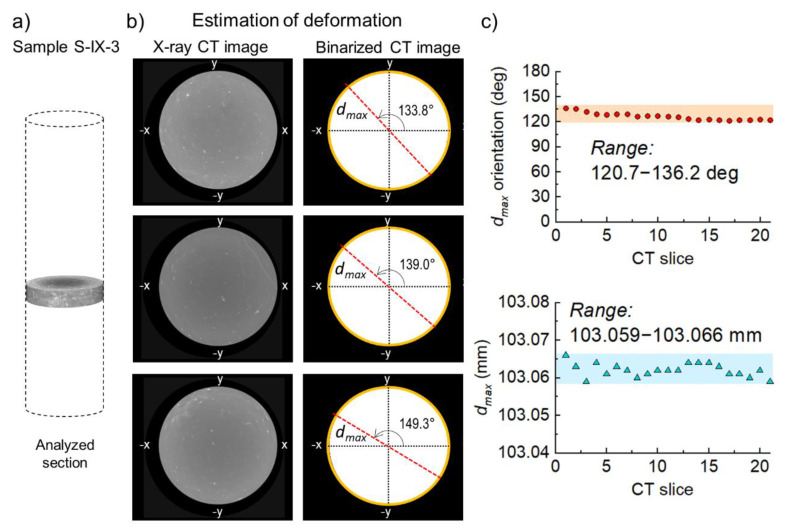
(**a**) X-ray 3D rendered section of sample S-IX-3. (**b**) Process of diametrical estimation using CT slices and ellipse fitting tools at three different heights. The images were first binarized, and later a best-fitting process allowed obtaining maximum and minimum deformations and their orientations. (**c**) The results of *d_max_* orientation and *d_max_* values for the analyzed CT cross sections fell into relatively small ranges.

The diametrical deformation measurements on core S-IX-3 were carried out using a section of CT images (Figure 4). The maximum deformation of *d_max_* returned several similar values (Figure 4c bottom). For example, *d_max_* ranged from 103.059 mm to 103.066 mm, while *d_min_* ranged from 103.022 mm to 103.027 mm. Kwon et al. [17] estimated values of elastic modulus and Poisson’s ratio for these Pohang rock cores to be 33.5 GPa and 0.21 GPa, respectively. By combining these results with the range of diametrical deformations using Equation (1), a stress difference ranging from 8.6 MPa to 17.2 MPa was obtained. On the other hand, the mean values for *d_max_* and *d_min_* were 103.062 mm and 103.0125 mm, respectively, and therefore the stress difference based on mean values was 13.3 MPa.

The mean *S_Hmax_* measured from well EXP-1 (located near Pohang EGS site) through hydraulic fracturing tests was 22.4 MPa, and with a *S_Hmax_/S_hmin_* ratio of 1.7, the result for *S_hmin_* was found to be 13.18 MPa [12]. This provides a stress difference of 9.22 MPa, a value that falls into the stress difference range given above (8.6 MPa to 17.2 MPa), although it is lower than the mean of 13.3 MPa. Alternatively, the Korean Government Commission reported a stress difference of 123 MPa and 110 MPa for the preferred model and regional stress models [22], values estimated for Pohang EGS site that are distinctively higher that those stress difference ranges presented above and estimated with the DCDA method.

### 3.2. Surface Roughness Assessment of Closed Joing CJ-2

**Figure 5:**
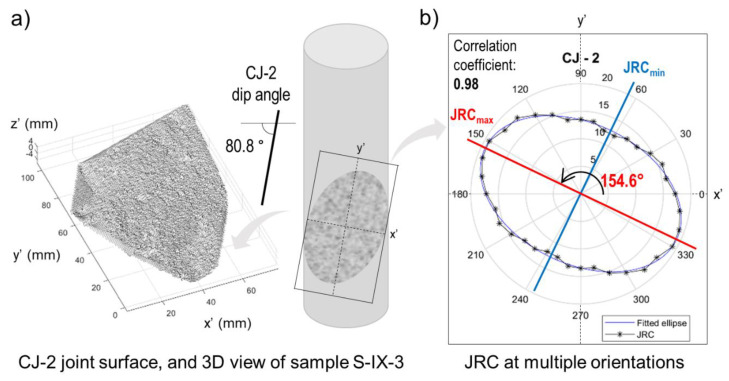
(**a**) Wireframe representation of CJ-2 joint on relative plane x′-y′, and schematic of sample S-IX-3 showing joint CJ-2 location. (**b**) Results of *JRC* measurements carried out using multiple parallel cross sections at 10 degrees increments. The mean value per orientation was then plotted in the polar diagram. To refine the anisotropy estimation, an ellipse fitting process was implemented (correlation coefficient 0.98), from which the orientations of *JRC_max_* and *JRC_min_* were obtained on the joint relative plane (dip angle 80.8°).

The results of the surface roughness re-assessment of closed joint CJ-2 are shown in Figure 5. As explained above, sample S-IX-3 contains a closed joint with a dip angle of 80.8°. Following Diaz et al. [18], multiple *JRC* measurements with multiple parallel cross sections per orientation were carried out. Then, the results for each orientation were averaged to return one value. This process was repeated every 10°, starting at 0° and finishing at 350°. The *JRC_max_* and *JRC_min_* orientation results were 154.6° and 64.6°, respectively, and were measured counter clockwise from a horizontal reference line. Moreover, *JRC_max_* and *JRC_min_* orientations were perpendicular to each other on the CJ-2 relative plane.

### 3.3. A Possible Correlation between Roughness Anisotropy and Stress Orientation

**Figure 6:**
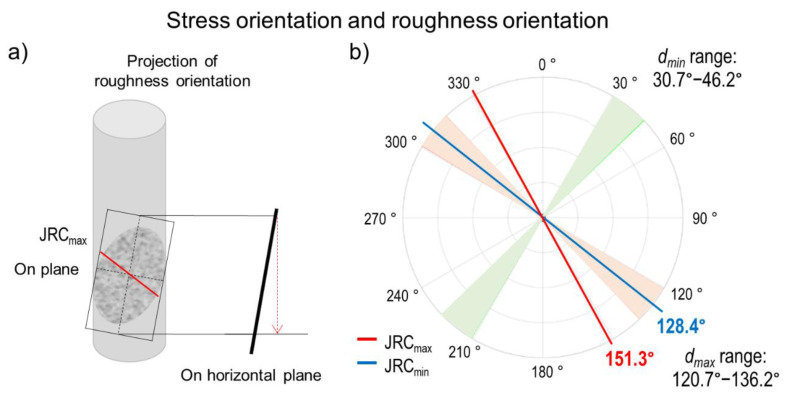
(**a**) Horizontal projection of *JRC_max_* and *JRC_min_* orientations on closed joint CJ-2 (dip angle of 80.8°). (**b**) Comparison of *d_max_* and *d_min_* orientations with the horizontal projections of *JRC_max_* and *JRC_min_* orientations. As was shown in Figure 4c, the *d_max_* orientation was not a single value, but instead ranged from 120.7°–136.2° (red area), with the orientations for *d_min_* 90° apart(green area). Later, the horizontal projections of *JRC_max_* and *JRC_min_* orientations were added, and an alignment between *JRC_min_* and *d_max_* was observed.

The possibility of a relation between stresses and roughness of joints motivated us to compare the orientation of horizontal stresses and the orientation of maximum roughness values in the S-IX-3 core sample. Similar to the results for *S_Hmax_* and *S_hmin_* using the DCDA method, the orientations of the horizontal stresses also showed a range of values, although they were perpendicular to each other. In order to compare the orientation of the stresses and the orientations of *JRC_max_* and *JRC_min_* in the same horizontal plane, the orientations of *JRC_max_* and *JRC_min_* were projected from the plane of the joint (dip of 80.0°) to the horizontal plane. These projections as well as the orientations of *d_max_* and *d_min_* are shown in Figure 6. The results showed how *JRC_min_* aligned well with the orientation of *d_max_* (*S_Hmax_*), as it felt into the range of this parameter. Although more comparisons with multiple cores are desirable, this apparent alignment opens up the discussion of a possible relation between the orientation of the maximum stress and the orientation of the lowest roughness value.

The shear strength anisotropy of rock joints was pointed out by Huang et al. [26], in their experimental and analytical study with rock joint replicas tested under different normal stresses and shearing directions. They showed a *JRC* dependence on the shearing direction. A different study on stylolites demonstrated how the orientation of surface anisotropy on vertical tectonic stylolites coincided with the principal stress directions, and concluded that the surface anisotropy of tectonic stylolites can be related to the forming stresses [27].

The role of stresses on rock joint roughness has also been mentioned by Barton et al. [28], as they showed how *JRC* increases as the stress anisotropy decreases. They aslo noted how smoother joints with lower *JRC* were formed under higher stress difference. However, they did not estimate the effect of the stress difference on the anisotropy of joint roughness. Considering the *JRC* dependence on the shearing direction, the alignment between *S_Hmax_* orientation and *JRC_min_* orientation shown here could be derived as a direct result of the shearing direction guided by the maximum stress at the time of formation of the rock joint. Then, the alignment of the maximum stress orientation and the orientation of lower roughness opens up this discussion to focus on how the joint roughness could carry information about the orientation of the forming stresses, although further studies with a larger number of samples are desirable.

### 3.4. Drawbacks and Limitations

One of the drawbacks of the DCDA method is the requirement to extract the cores perpendicular to the studied stresses. In other words, if the coring direction is slightly deviated from the true perpendicular orientation, this small difference can affect the estimations of the stress difference. Moreover, if only one coring orientation is considered, for example the vertical, then only the stress difference between maximum and minimum horizontal stresses can be obtained. Then, it is still necessary to know the magnitude of one of the stresses to compute the other using the obtained stress difference. However, to overcome the issue of the stress magnitude and orientation, Funato and Ito [9] have suggested to obtain at least three cores from different directions.

The DCDA method was validated by Funato and Ito [9] using multiple laboratory tests and with different types of rock samples. For those tests, the horizontal stresses acting during coring were known, and therefore it was possible to estimate the error between the estimated and the actual stress differences (error of ± 0.87 MPa). However, in this study, it was not possible to estimate the error since the true stress values are unknown. Nevertheless, the stress difference reported by another study that used a different technique fell into the stress difference range obtained here and discussed in Section 3.1.

Finally, the big advantage that this method could offer is economical, especially compared to traditional methods for stress estimation such as hydraulic fracturing. This could positively affect many industries, especially geothermal development due to its need for deeper drilling where stresses play an important role.

## 4. Conclusions

Estimation of in-situ stresses based on the diametrical deformation of a rock core was carried out. The diametrical core deformation analysis (DCDA) method was used to estimate the stress difference based on the anisotropic expansion of rock cores diameter after stress relief assuming elastic deformation. The granodiorite rock core (100 mm in diameters) used in this study came from an Enhanced Geothermal System project in South Korea, and it was extracted at a 4.2 km depth. A roughness analysis on the closed joint crossing the core sample has shown a roughness anisotropy with an orientation of the maximum value at 154.6° measured counterclockwise from the horizontal joint plane on the rock. On the other hand, the diametrical core deformation was calculated using X-ray CT images, and the DCDA method returned an average stress difference of 13.3 MPa, a value that was closer to estimations through hydraulic fracturing tests in a nearby well. Finally, the superposition of stresses and roughness revealed a correlation between the orientation of the maximum horizontal stress and the orientation of the minimum *JRC*, opening up a possibility for tracking stress orientations based on rock joint roughness anisotropy.

## Figures and Tables

**Table 1:** Process followed to estimate stress difference and joint roughness for sample S-IX-3.

No.	Step	Description
Step 1	Site recovery	Rock cores recovery at a depth of around 4.2 km.
Step 2	X-ray CT scanning	Scanning of sample S-IX-3, and creation of CT cross sections and point cloud data of the sample surface.
Step 3	Differential stress	Horizontal stress difference estimation using the DCDA method.
Step 4	Joint roughness	Calculation of joint roughness anisotropy of closed joint CJ-2, which crosses through sample S-IX-3.
Step 5	Result discussion	Discussion on the orientations of S_Hmax_ and maximum roughness.
